# Sirtuin five knockdown induced the ferroptosis of hepatic stellate cells in hepatic fibrosis progression through de-succinylation modification of GPX4

**DOI:** 10.1016/j.jbc.2026.113366

**Published:** 2026-07-24

**Authors:** Jun Zhou, Jinjuan Zhang, Shan Zheng, Jiahong Zhu, Kelei Wang, Xiumei Guo

**Affiliations:** 1 College of Basic Medicine, Guiyang Healthcare Vocational University, Guiyang, Guizhou, China; 2 School of Basic Medicine, Guizhou Medical University, Guiyang, Guizhou, China; 3 College of Pharmacy, Guiyang Healthcare Vocational University, Guiyang, Guizhou, China

**Keywords:** hepatic fibrosis, SIRT5, ferroptosis, succinylation, GPX4

## Abstract

Hepatic fibrosis results from persistent pathological damage and repair following various chronic liver injuries. In this study, we explored the specific mechanism of sirtuin 5 (SIRT5) in the development of hepatic fibrosis. Transforming growth factor-beta 1 (TGF-β1)-treated LX-2 cells and CCl_4_-treated mice were used to establish hepatic fibrosis models *in vitro* and *in vivo*. Cell viability was assessed using the CCK-8 assay, and ferroptosis-related indicators were measured with commercial kits. Protein expression and succinylation levels were analyzed by Western blot. Liver tissue structure in fibrotic mice was evaluated using hematoxylin and eosin (H&E), Masson, and Sirius Red staining. SIRT5 expression and global succinylation levels were elevated in TGF-β1-treated LX-2 cells. Knockdown of SIRT5 reduced cell viability and glutathione (GSH) levels while increasing malondialdehyde (MDA), iron, and reactive oxygen species (ROS) levels in these cells. Furthermore, SIRT5 knockdown enhanced the succinylation of glutathione peroxidase 4 (GPX4) and decreased GPX4 protein stability and expression. *In vivo* experiments showed that SIRT5 knockdown ameliorated liver structure in fibrotic mice by targeting GPX4 expression. Our study demonstrates that SIRT5 knockdown induces ferroptosis in TGF-β1-treated LX-2 cells by decreasing GPX4 protein levels *via* desuccinylation.

Hepatic fibrosis results from persistent pathological damage and repair induced by various chronic liver injuries, including viral infection, long-term alcohol consumption, metabolic disorders, and autoimmune diseases (1). Severe hepatic fibrosis may even progress to cirrhosis or liver cancer (2, 3). Therefore, effective prevention and reversal of hepatic fibrosis have become global medical challenges. Current research indicates that activation of hepatic stellate cells (HSCs) is a central event in the onset and progression of hepatic fibrosis (4). Upon liver injury, quiescent HSCs become activated, proliferate continuously, and secrete large amounts of extracellular matrix. Concurrently, various inflammatory factors and chemokines are released, affecting hepatocytes and Kupffer cells and further exacerbating the pathological state of the liver (5, 6). Consequently, targeted inhibition of HSC activation is a key approach for ameliorating and treating hepatic fibrosis.

In recent years, numerous studies have shown that, compared with normal liver tissue, iron ion levels and lipid peroxidation are significantly increased in fibrotic liver tissue, suggesting that ferroptosis may contribute to the progression of hepatic fibrosis (7, 8, 9). In 2012, Dr Brent R. Stockwell’s team proposed ferroptosis as a novel form of programmed cell death that is iron-dependent and distinct from apoptosis and autophagy (10). This unique cell death pathway, driven by iron-dependent phospholipid peroxidation, is regulated by multiple cellular metabolic processes, including redox homeostasis, iron ion levels, mitochondrial activity, and the metabolism of amino acids, lipids, and sugars, as well as by various disease-related signaling pathways (11). The essence of ferroptosis is the depletion of glutathione (GSH), a decrease in glutathione peroxidase 4 (GPX4) activity, and the failure to metabolize lipid peroxides *via* the GPX4-catalyzed glutathione reaction, which disrupts cell membrane integrity and leads to cell death (12). Recent studies have demonstrated that HSCs store abundant iron ions, and ferroptosis can influence the progression of hepatic fibrosis by regulating iron ion content and lipid peroxidation levels in HSCs (13, 14). Thus, targeting ferroptosis in HSCs may represent a novel therapeutic strategy for hepatic fibrosis.

Protein post-translational modifications (PTMs) are widespread in prokaryotic and eukaryotic organisms, participating in various life activities and playing important roles (15). Common PTMs include phosphorylation, acetylation, methylation, ubiquitination, methylmalonylation, and succinylation (16). Succinylation is relatively conserved during biological evolution and is involved in almost all biological processes. Compared with other PTMs, succinylation induces more pronounced changes in protein physicochemical properties and functions (17). With the continuous advancement of mass spectrometry technology, research has revealed that succinylation participates in numerous biological processes, such as oxidation, metabolic regulation, and signal transduction, and is closely associated with the development of various human diseases (18). Among the Sirtuin family proteins, which are frequently implicated in succinylation modification in eukaryotes, sirtuin 5 (SIRT5) is currently recognized as an enzyme that negatively regulates succinylation (19). SIRT5 is primarily located in mitochondria but is also found in the cytoplasm and nucleus. Owing to its unique structure and specific amino acid recognition, SIRT5 exhibits strong desuccinylation activity (20). A previous study demonstrated that high SIRT5 levels reduced reactive oxygen species (ROS) levels and increased GSH and GPX4 levels by regulating the succinylation of isocitrate dehydrogenase 2 (IDH2) and glucose-6-phosphate dehydrogenase (G6PD), thereby enhancing cellular antioxidant defense and inhibiting ferroptosis (21). However, the role of SIRT5 in hepatic fibrosis remains unclear.

Therefore, in this study, we demonstrated that SIRT5 is upregulated in transforming growth factor-beta 1 (TGF-β1)-treated HSCs. Knockdown of SIRT5 enhanced GPX4 succinylation and decreased GPX4 protein expression, which subsequently induced HSC ferroptosis. Our findings suggest that dysregulation of SIRT5 expression may be a key factor in the development of hepatic fibrosis.

## Results

### TGF-β1 decreased the succinylation levels of LX-2 cells

**Figure 1:**
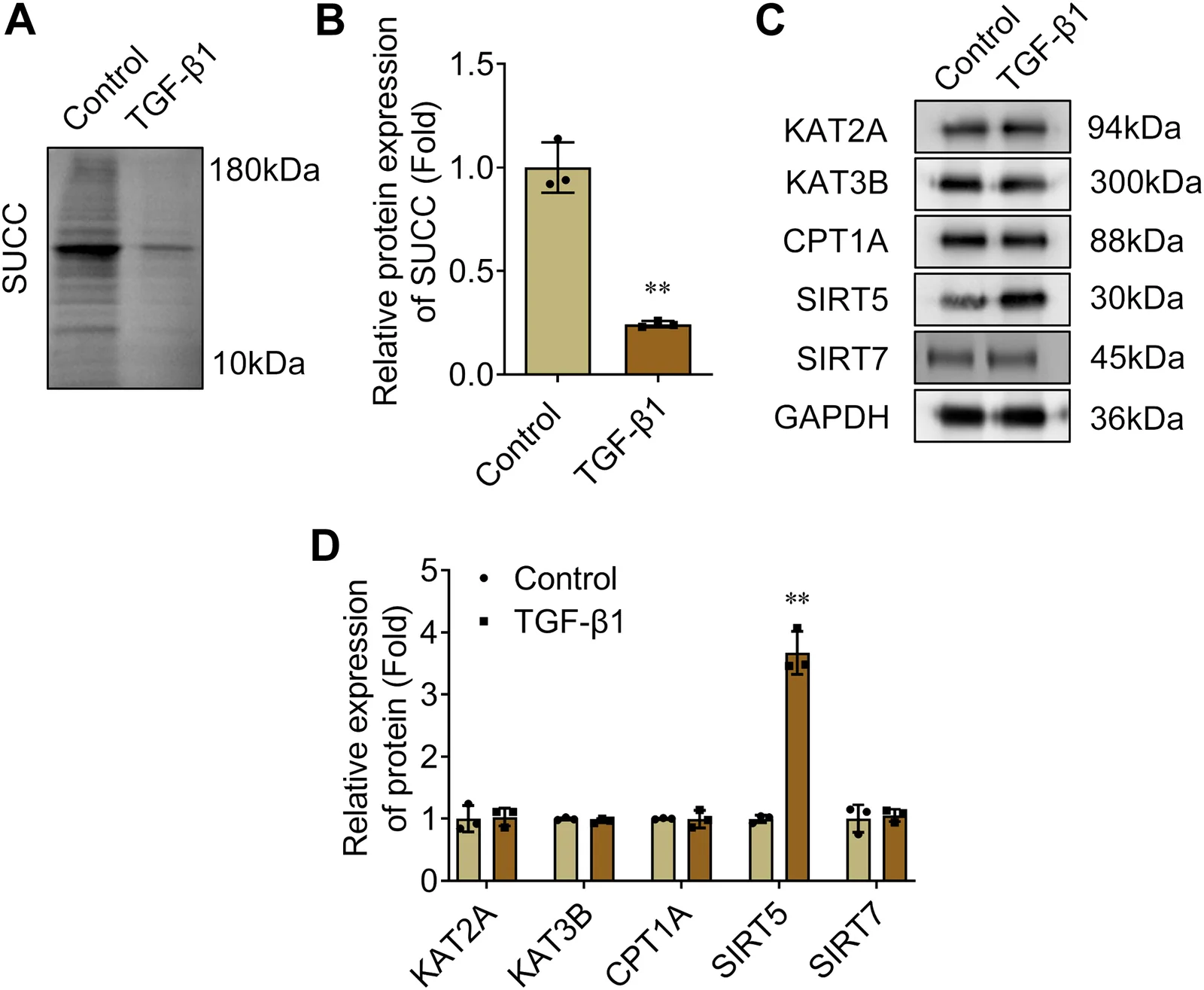
**TGF-β1 decreased the succinylation levels of LX-2 cells.** The LX-2 cells were treated with 5 ng/ml TGF-β1 for 24 h. The global succinylation (*A* and *B*) of the cells and the expression of succinylation-related proteins (*C* and *D*) were detected by Western blot. Each experiment has three biological replicates and three technical replicates. One-way ANOVA followed by tukey was applied to assess differences between multiple groups.

TGF-β1 was used to treat LX-2 cells to establish an *in vitro* hepatic fibrosis model. We found that global succinylation levels in LX-2 cells were significantly decreased after TGF-β1 treatment (Fig. 1, *A* and *B*). Ponceau S staining of the membrane is shown in Fig. S1 as a loading control for the global succinylation Western blot. We further analyzed the expression of succinylation-related proteins in TGF-β1-treated LX-2 cells. TGF-β1 treatment significantly increased SIRT5 protein levels in LX-2 cells but did not affect the expression of other tested proteins (Fig. 1, *C* and *D*). These results indicate that the decrease in succinylation levels in TGF-β1-treated LX-2 cells may be induced by the increase in SIRT5 protein expression.

### SIRT5 knockdown inhibited LX-2 cell activation by inducing ferroptosis

**Figure 2:**
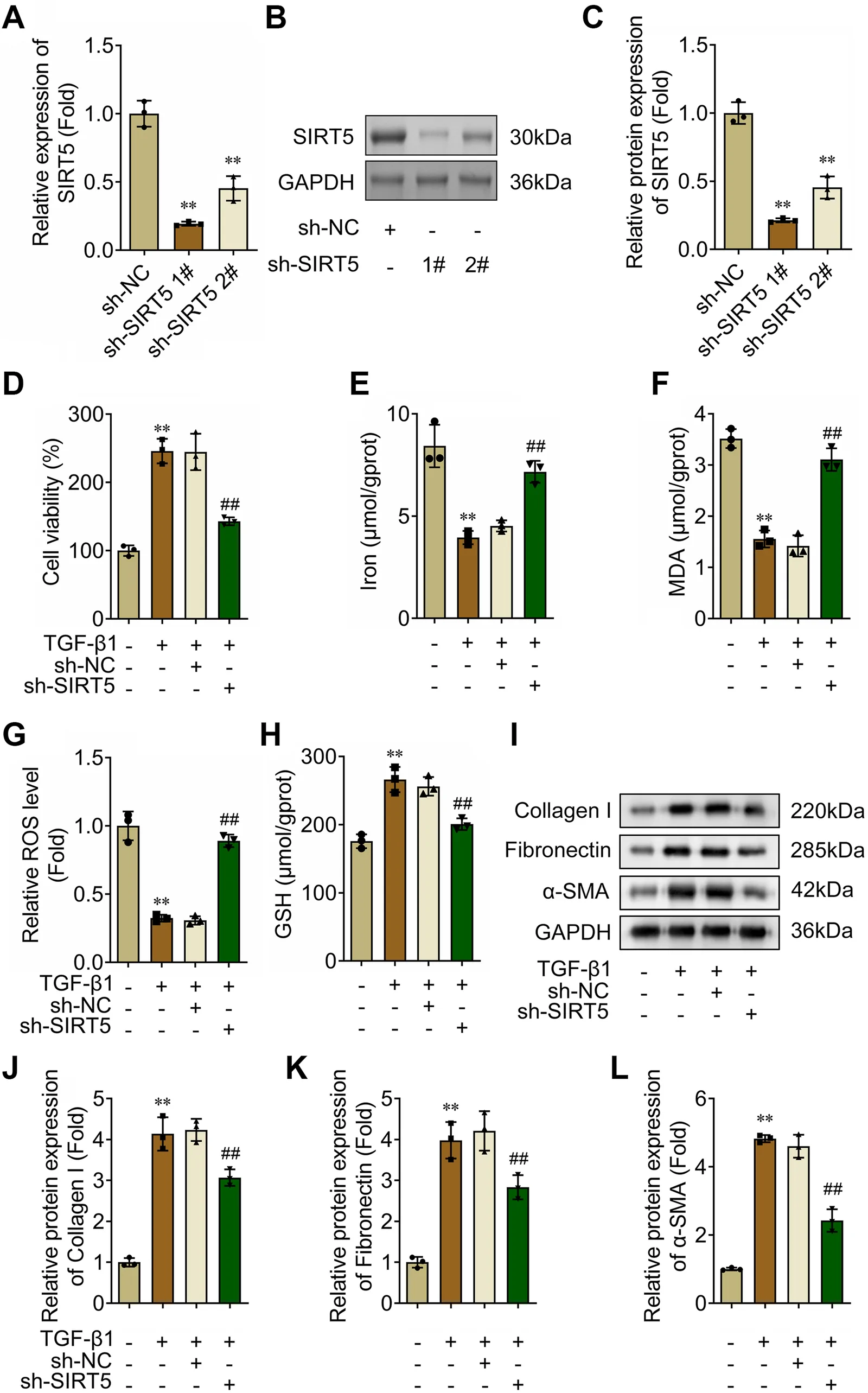
**SIRT5 knockdown inhibited the activation of LX-2 cell by inducing the ferroptosis.** After sh-SIRT5 1# and sh-SIRT5 2# transfection, the mRNA and protein levels of SIRT5 in the LX-2 cells were detected by RT-qPCR (*A*) and Western blot (*B* and *C*) assays. Then, the LX-2 cells were treated with 5 ng/ml TGF-β1 and transfected with sh-SIRT5 1#. *D*, Cell viability was detected by CCK-8 assay. The Iron (*E*), MDA (*F*), ROS (*G*), and GSH (*H*) levels were detected by commercial kits. *I-L*, the protein levels of Collagen I, fibronectin, and α-SMA were detected by Western blot. ∗∗*p* < 0.01 *versus* Control group. ##*p* < 0.01 *versus* TGF-β1+sh-NC group. Each experiment has three biological replicates and three technical replicates. One-way ANOVA followed by Tukey was applied to assess differences between multiple groups.

We further knocked down SIRT5 expression in LX-2 cells by transfecting sh-SIRT5. After sh-SIRT5 transfection, both the mRNA (Fig. 2*A*) and protein (Fig. 2, *B* and *C*) levels of SIRT5 were significantly decreased. The CCK-8 assay showed that TGF-β1 treatment significantly increased the viability of LX-2 cells, whereas SIRT5 knockdown decreased cell viability (Fig. 2*D*). In addition, TGF-β1 treatment significantly reduced iron (Fig. 2*E*), malondialdehyde (MDA) (Fig. 2*F*), and ROS (Fig. 2*G*) levels and increased GSH levels (Fig. 2*H*) in LX-2 cells. SIRT5 knockdown significantly increased iron, MDA, and ROS levels and decreased GSH levels in TGF-β1-treated LX-2 cells (Fig. 2, *E*–*H*). Furthermore, the protein levels of Collagen I, fibronectin, and α-SMA were significantly increased after TGF-β1 treatment, and SIRT5 knockdown significantly reduced these levels (Fig. 2, *I*–*L*). We also performed these experiments using a second sh-SIRT5 sequence, which confirmed that SIRT5 knockdown induces ferroptosis and inhibits LX-2 cell activation (Fig. S2). Together, these results demonstrate that SIRT5 knockdown suppresses LX-2 cell activation.

### SIRT5 knockdown inhibited hepatic fibrosis *in vivo*

**Figure 3:**
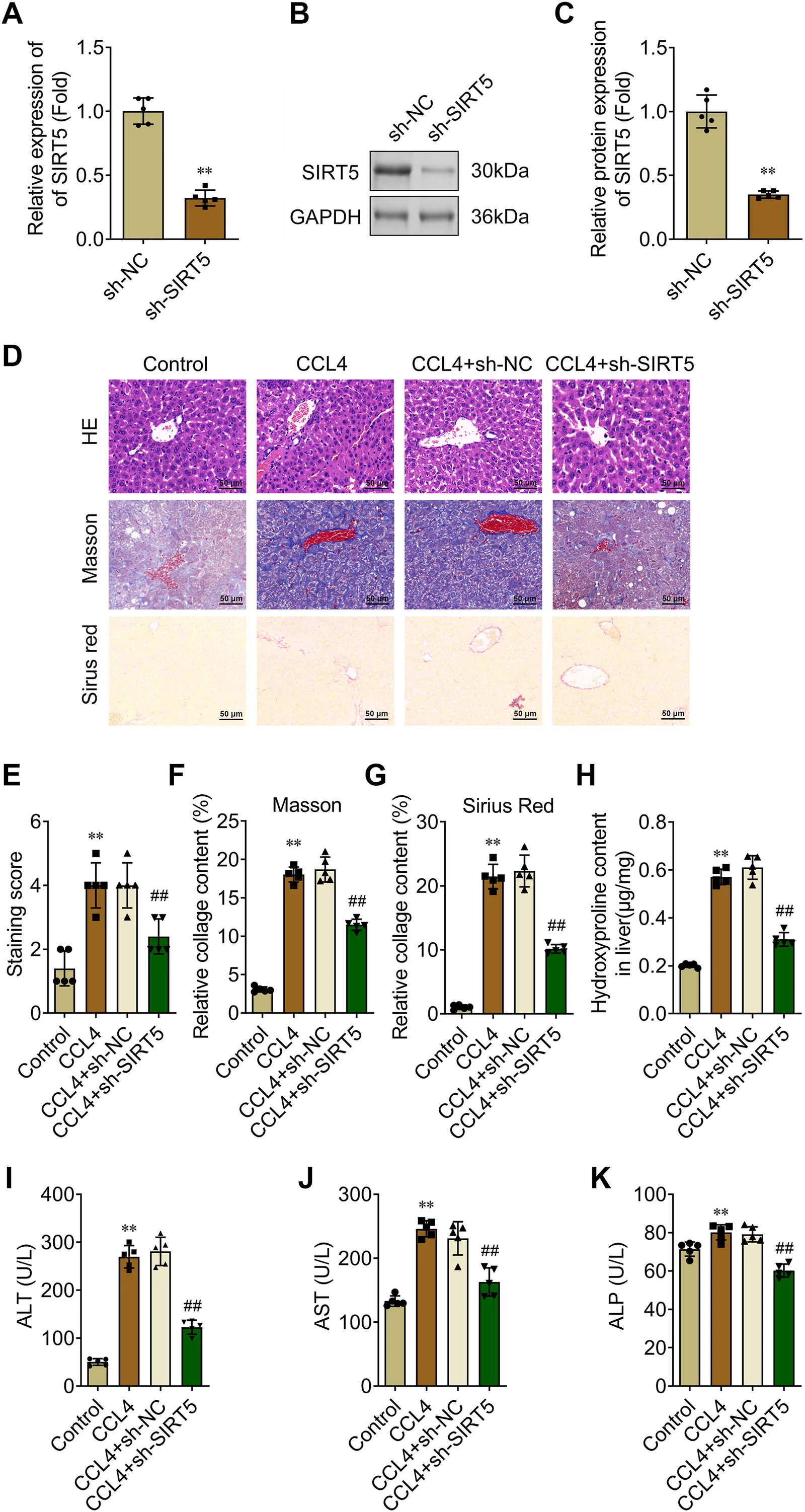
**SIRT5 knockdown inhibited hepatic fibrosis *in vivo*.** ShSIRT5 derived from mice was used for *in vivo* experiments. The SIRT5 levels in the liver of hepatic fibrosis mice were detected by RT-qPCR (*A*) and Western blot assay (*B* and *C*). *D-G*, the H&E, Masson and Sirius Red staining of the liver samples in hepatic fibrosis mice, scale bar = 50 μM. *H*, the hydroxyproline levels in the liver of hepatic fibrosis mice were detected by commercial kits. The ALT (*I*), AST (*J*), and ALP (*K*) levels in the serum of the hepatic fibrosis mice were detected by commercial kits.∗∗*p* < 0.01 *versus* Control group. ##*p* < 0.01 *versus* CCl_4_+sh-NC group. Each experiment has six biological replicates and three technical replicates. One-way ANOVA followed by Tukey was applied to assess differences between multiple groups.

We next explored the role of SIRT5 in hepatic fibrosis progression using a mouse model. After sh-SIRT5 injection, the mRNA (Fig. 3*A*) and protein (Fig. 3, *B* and *C*) levels of SIRT5 were significantly decreased. Haematoxylin and eosin (H&E), Masson, and Sirius Red staining revealed hepatocyte degeneration, inflammatory cell infiltration, fibrous scarring, and collagen deposition in the liver samples of the CCl_4_ and CCl_4_ + sh-NC groups, whereas these changes were markedly alleviated after SIRT5 knockdown (Fig. 3, *D*–*G*). Moreover, the elevated hydroxyproline levels in the livers of CCl_4_-treated mice were reduced following SIRT5 knockdown (Fig. 3*H*). Serum levels of liver injury-related indicators (AST, ALT, and ALP) were significantly increased in CCl_4_ and CCl_4_ + sh-NC-treated mice, and SIRT5 knockdown significantly decreased them (Fig. 3, *I*–*K*).

### SIRT5 knockdown decreased GPX4 levels by increasing GPX4 succinylation

**Figure 4:**
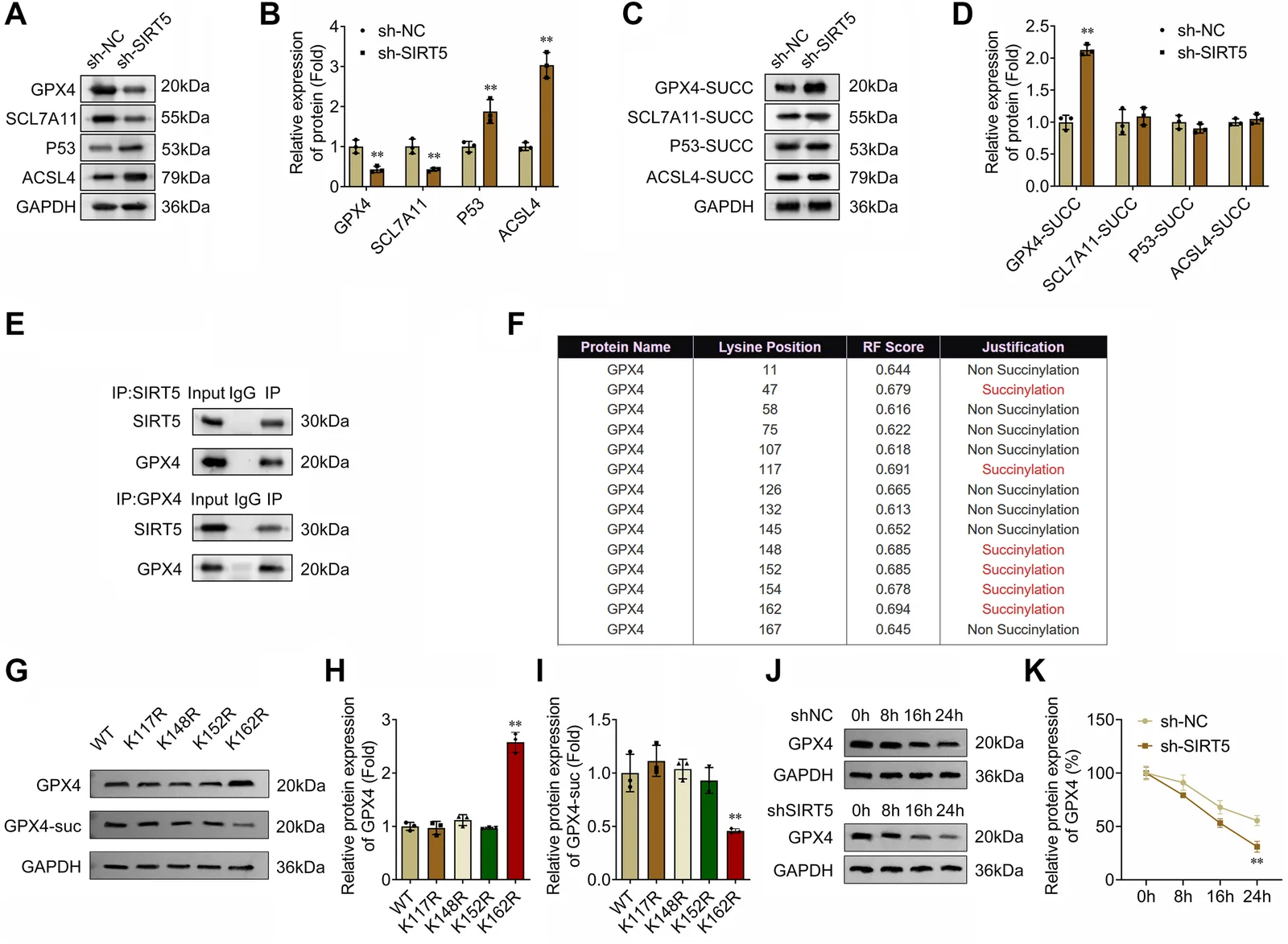
**SIRT5 knockdown decreased the GPX4 levels through increasing the succinylation levels of GPX4.** The LX-2 cells were treated with 5 ng/ml TGF-β1 and transfected with sh-SIRT5. Then the protein levels (*A* and *B*) and succinylation levels (*C* and *D*) of GPX4, SLC7A11, P53 and ACSL4 were detected by Western blot. *E*, the relationship between SIRT5 and GPX4 was demonstrated by Co-IP assay. *F*, GPSuc website was used for prediction of succinylation sites of GPX4. *G-I*, the protein and succinylation levels of GPX4 were detected by Western blot after succinylation sites mutation. *J and K*, the protein stability of GPX4 was detected by Western blot after SIRT5 knockdown. ∗∗*p* < 0.01 *versus* sh-NC or WT group. Each experiment has three biological replicates and three technical replicates. One-way ANOVA followed by tukey was applied to assess differences between multiple groups.

We next investigated the underlying mechanism of SIRT5. SIRT5 knockdown decreased the protein levels of GPX4 and SLC7A11 and increased those of p53 and ACSL4 in TGF-β1-treated LX-2 cells (Fig. 4, *A* and *B*). Succinylation analysis showed that SIRT5 knockdown significantly increased GPX4 succinylation but did not affect the succinylation of SLC7A11, p53, or ACSL4 (Fig. 4, *C* and *D*). Co-IP assays revealed an interaction between SIRT5 and GPX4 (Fig. 4*E*). Using the GPSuc website, we predicted succinylation sites of GPX4, including K47, K117, K148, K152, K154, and K162 (Fig. 4*F*). Based on the modification scores, we selected the top four sites (K117R, K148R, K152R, K162R) for mutation. After the K162 mutation, GPX4 succinylation was decreased, suggesting that SIRT5 may regulate GPX4 succinylation at the K162 residue (Fig. 4, *G*–*I*). Finally, SIRT5 knockdown accelerated the protein degradation of GPX4 (Fig. 4, *J* and *K*). These results indicate that SIRT5 knockdown decreases GPX4 protein levels by increasing GPX4 succinylation.

### GPX4 overexpression reversed the effects of SIRT5 knockdown in TGF-β1-treated LX-2 cells

**Figure 5:**
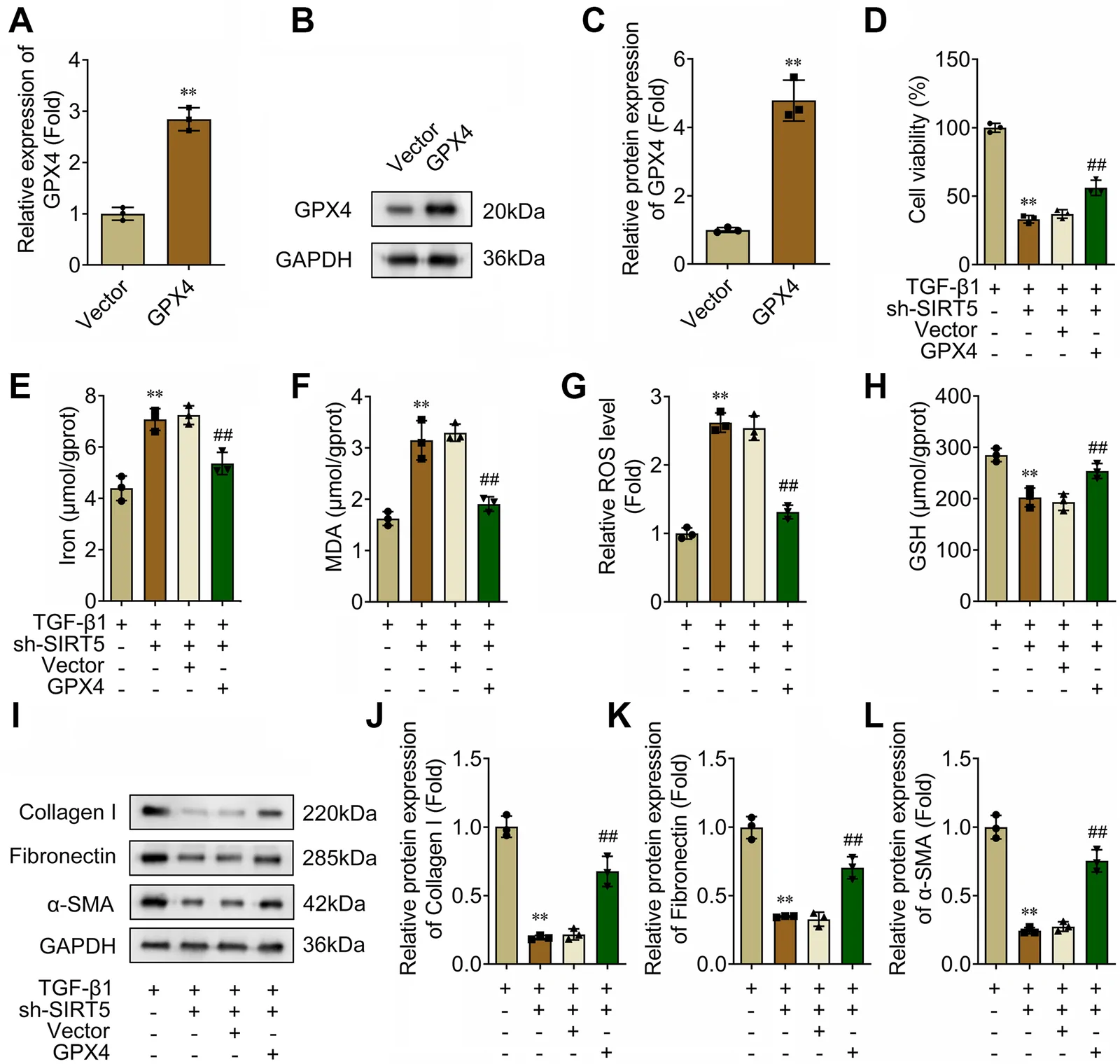
**GPX4 overexpression reversed the role of SIRT5 knockdown in the TGF-β1-treated LX-2 cells.** The LX-2 cells were treated with 5 ng/ml TGF-β1 and transfected with sh-SIRT5 and GPX4. After GPX4 transfection, the mRNA and protein levels of GPX4 in the LX-2 cells were detected by RT-qPCR (*A*) and Western blot (*B* and *C*) assays. *D*, the cell viability was detected by CCK-8 assay. The Iron (*E*), MDA (*F*), ROS (*G*), and GSH (*H*) levels were detected by commercial kits. *I-L*, the protein levels of Collagen I, fibronectin, and α-SMA were detected by Western blot. ∗∗*p* < 0.01 *versus* TGF-β1 group. ##*p* < 0.01 *versus* TGF-β1+sh-SIRT5+vector group. Each experiment has three biological replicates and three technical replicates. One-way ANOVA followed by tukey was applied to assess differences between multiple groups.

To further investigate the relationship between SIRT5 and GPX4, we overexpressed GPX4 in sh-SIRT5-and TGF-β1-treated LX-2 cells by transfecting oe-GPX4. After oe-GPX4 transfection, GPX4 mRNA (Fig. 5*A*) and protein (Fig. 5, *B* and *C*) levels were significantly increased. In sh-SIRT5-and TGF-β1-treated LX-2 cells, GPX4 overexpression significantly increased cell viability (Fig. 5*D*), decreased iron, MDA, and ROS levels (Fig. 5, *E*–*G*), and increased GSH levels (Fig. 5*H*). Additionally, GPX4 overexpression significantly increased the protein levels of Collagen I, fibronectin, and α-SMA in sh-SIRT5-and TGF-β1-treated LX-2 cells (Fig. 5, *I*–*L*). In SIRT5-overexpressing LX-2 cells, GPX4 with the K162 succinylation site mutation showed significantly weaker promoting effects on ferroptosis and cell activation than wild-type GPX4 (Fig. S3). These results confirm that SIRT5 knockdown inhibits LX-2 cell activation by regulating GPX4 succinylation at K162.

### GPX4 overexpression reversed the effects of SIRT5 knockdown *in vivo*

**Figure 6:**
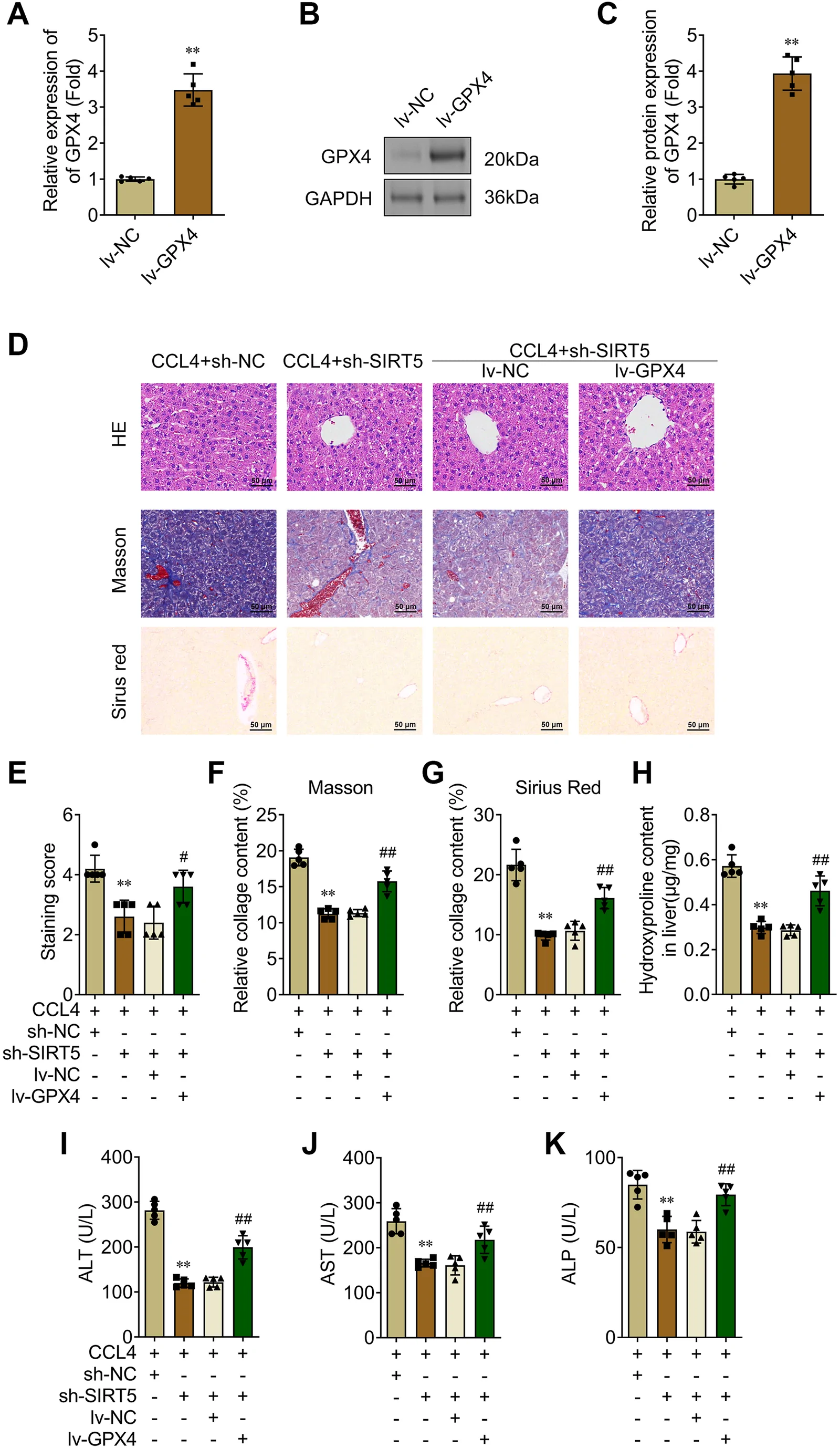
**SIRT5 knockdown inhibited hepatic fibrosis *in vivo*.** The GPX4 levels in the liver of hepatic fibrosis mice were detected by RT-qPCR (*A*) and Western blot assay (*B* and *C*). *D-G*, the H&E, Masson and Sirius Red staining of the liver samples in hepatic fibrosis mice, scale bar = 50 μM. *H*, the hydroxyproline levels in the liver of hepatic fibrosis mice were detected by commercial kits. The ALT (*I*), AST (*J*), and ALP (*K*) levels in the serum of the hepatic fibrosis mice were detected by commercial kits.∗∗*p* < 0.01 *versus* CCl_4_+sh-NC group. ##*p* < 0.01 *versus* CCl_4_+sh-SIRT5+lv-NC group. Each experiment has six biological replicates and three technical replicates. One-way ANOVA followed by Tukey was applied to assess differences between multiple groups.

Finally, we examined the role of GPX4 overexpression in hepatic fibrosis progression using a mouse model. After Lv-GPX4 injection, GPX4 mRNA (Fig. 6*A*) and protein (Fig. 6, *B* and *C*) levels were significantly increased. H&E, Masson, and Sirius Red staining showed that GPX4 overexpression attenuated the protective effects of SIRT5 knockdown against hepatocyte degeneration, inflammatory cell infiltration, fibrous scarring, and collagen deposition (Fig. 6, *D*–*G*). Furthermore, GPX4 overexpression also increased hydroxyproline levels in the liver (Fig. 6*H*) and the serum levels of AST, ALT, and ALP in CCl_4_-and sh-SIRT5-treated mice (Fig. 6, *I*–*K*).

## Discussion

In this study, we demonstrated that SIRT5 knockdown induces ferroptosis in TGF-β1-treated LX-2 cells. Mechanistically, SIRT5 knockdown decreased GPX4 protein levels by regulating the desuccinylation modification of GPX4. *In vivo* experiments indicated that SIRT5 knockdown inhibited the progression of hepatic fibrosis. Furthermore, GPX4 overexpression reversed the effects of SIRT5 knockdown both *in vivo* and *in vitro*.

The main pathological features of hepatic fibrosis include activation of hepatic stellate cells (HSCs), excessive deposition of extracellular matrix (ECM) in the liver, and abnormal proliferation of fibrous connective tissue in the portal area, which together disrupt the normal structure and physiological function of the liver (22, 23, 24). Therefore, inhibiting HSC activation and proliferation and inducing HSC death represent effective strategies for treating liver fibrosis. Sui *et al.* (25) found that magnesium isoglycyrrhetinate induced ferroptosis of HSCs in a rat model of hepatic fibrosis by upregulating HO-1 levels and promoting iron accumulation and lipid peroxidation. Notably, the anti-fibrotic effect of magnesium isoglycyrrhetinate was abolished by the ferroptosis inhibitor ferrostatin-1. SIRT5, a classic desuccinylation enzyme, has been reported to participate in various liver diseases. For example, Zhang *et al.* (26) demonstrated that SIRT5 knockdown increased ROS levels and aggravated lipid accumulation in hepatocytes, thereby counteracting the therapeutic effect of 2,3,5,4′-tetrahydroxy-stilbene-2-O-β-D-glucoside in non-alcoholic fatty liver disease. In addition, Zhou *et al.* (27) found that SIRT5 levels were significantly increased in the liver after myocardial infarction and confirmed that SIRT5 may exert a cardioprotective effect in response to acute ischemia through liver-cardiac crosstalk. These studies indicate that the role of SIRT5 in cellular growth and development is context-dependent, acting as a double-edged sword depending on the disease and cell type. In the present study, we found that SIRT5 was upregulated in TGF-β1-treated LX-2 cells. Knockdown of SIRT5 in these cells inhibited LX-2 cell activation and induced ferroptosis, manifested as decreased cell viability and increased levels of MDA, ROS, and iron. Furthermore, *in vivo* experiments showed that SIRT5 knockdown ameliorated the pathological morphology of liver tissue in fibrotic mice and reduced serum AST, ALT, and ALP levels. These results preliminarily suggest that SIRT5 knockdown may inhibit hepatic fibrosis progression by inducing ferroptosis in HSCs.

Many researches reported that SIRT5 can participate in inflammatory reactions, oxidative stress, and organ injury by regulating succinylation levels (28, 29). Rardin *et al.* (30)identified 1190 succinylation sites in the liver mitochondria and found 386 sites across 140 proteins were significantly hypersuccinylated after SIRT5 knockout. Nakagawa *et al.* (31) found that SIRT5 was localized in the mitochondrial matrix and interacted with phosphocarbamoyl synthetase one through regulating the succinylation. However, the substrate specificity of SIRT5 remains a subject of debate. For instance, a recent study demonstrated that SIRT5 does not influence lysine succinylation of peroxisomal proteins, suggesting that its desuccinylase activity is highly dependent on subcellular localization and protein context (32). In line with this notion, we found that while SIRT5 knockdown increased the succinylation level of GPX4, it did not affect the succinylation of other ferroptosis-related proteins such as SLC7A11, P53, and ACSL4. This indicates that the regulation of GPX4 by SIRT5 is relatively specific, rather than a general effect on all proteins involved in ferroptosis.

Furthermore, it is valuable to compare our findings with other known SIRT5 targets. For example, SIRT5 has been shown to desuccinylate and activate citrate synthase (CS), a key enzyme in the tricarboxylic acid (TCA) cycle, thereby regulating mitochondrial metabolism (33). Drawing a parallel, both GPX4 and CS are regulated by SIRT5-mediated desuccinylation to enhance their functional stability or activity. However, a key difference lies in the functional consequence: CS regulation affects metabolic flux, while GPX4 regulation directly controls the cellular ferroptosis defense system. This comparison highlights that SIRT5 acts as a versatile regulator that fine-tunes distinct biological processes—ranging from metabolism to cell death—by specifically modulating the succinylation status of key target proteins. Therefore, our study not only identifies GPX4 as a critical succinylation substrate of SIRT5 in hepatic fibrosis but also adds to the growing understanding of SIRT5's context-dependent substrate selectivity.

In conclusion, this study demonstrated that SIRT5 was upregulated in the TGF-β1-treated LX-2 cells. SIRT5 knockdown inhibited the activation of HSCs and relieved the hepatic fibrosis progression *in vivo*. Mechanistically, SIRT5 regulated the GPX4 expression levels through desuccinylation modification. Our research provided a new method for targeted treatment of hepatic fibrosis in future.

## Experimental procedures

### Experimental period

This study was conducted from January 1, 2023, to July 1, 2023. The animal experimental protocol was approved by the Ethics Committee of Guiyang Healthcare Vocational University (Approval No. 2023101, dated August 15, 2023). This study was conducted in full compliance with the ARRIVE guidelines 2.0 (Animal Research: Reporting of In Vivo Experiments) for reporting animal research. All animal experiments were performed in accordance with the National Institutes of Health Guide for the Care and Use of Laboratory Animals (eighth edition, 2011) and the relevant guidelines and regulations of China.

### Cell culture

Human hepatic stellate cells (LX-2, Catalog No. GNHu58) were purchased from the Chinese Academy of Medical Sciences. LX-2 cells were authenticated by short tandem repeat (STR) profiling and tested negative for *mycoplasma* contamination. All cells were cultured in DMEM supplemented with 10% fetal calf serum for 24 h. Subsequently, the cells were collected and treated with 5 ng/ml TGF-β1 for 24 h to establish an *in vitro* hepatic fibrosis model.

### Cell transfection

For SIRT5 knockdown, specific short hairpin RNA targeting SIRT5 (sh-SIRT5) and non-specific shRNA (sh-NC) were designed and provided by GenePharma. For GPX4 overexpression, pcDNA3.1-GPX4 overexpression plasmids (oe-GPX4) and negative control empty vectors were also obtained from GenePharma. LX-2 cells were transfected with the indicated shRNAs and vectors using Lipofectamine 3000 reagent. The sequences of sh-SIRT5 were as follows:

sh-SIRT5 #1: 5′-GCGAGAAACCCUACUACAACU-3′

sh-SIRT5 #2: 5′-CGAAUGGCGACCUGUAUUUCC-3′

sh-NC: 5′-UUCUCCGAACGUGUCACGU-3′

### Real-time quantitative PCR (RT-qPCR)

Total RNA was extracted using TRIzol reagent (Invitrogen). After centrifugation according to the manufacturer’s instructions, the supernatant was discarded, and the RNA pellet was dissolved in a 60 °C water bath. Total RNA (1 μg) was added to a microcentrifuge tube, incubated at 70 °C for 10 min, briefly centrifuged, and then placed on ice. A 20 μl reaction system was established. The reaction mixture was incubated at 42 °C for 15 min (an additional incubation step at this temperature facilitates primer extension, ensuring that primers remain hybridized when the temperature reaches 42 °C). The sample was then heated at 95 °C for 5 min and placed at 0 to 5 °C for 5 min qPCR data were analyzed using the 2^−ΔΔCT^ method. The primer sequences (5′→3′) were as follows:

SIRT5: Forward GCCATAGCCGAGTGTGAGAC, Reverse CAACTCCACAAGAGGTACATCG.

GPX4: Forward GAGGCAAGACCGAAGTAAACTAC, Reverse CCGAACTGGTTACACGGGAA.

GAPDH: Forward TGTGGGCATCAATGGATTTGG, Reverse ACACCATGTATTCCGGGTCAAT.

### Cell Counting Kit-8 (CCK-8) assay

Cell viability of LX-2 cells was assessed using the CCK-8 assay (Cat. CK04, Dojindo) according to the manufacturer’s instructions. After transfection and various treatments, LX-2 cells were seeded into 96-well plates and incubated with 10 μl of CCK-8 solution. Following further incubation at 37°C in a 5% CO_2_ atmosphere for 2 h, the absorbance was measured at 450 nm using a microplate reader (BMG Labtech).

### Determination of ferroptosis-related indexes

Malondialdehyde (MDA, Cat. A003-1-2), reactive oxygen species (ROS, Cat. E004-1-1), and glutathione (GSH, Cat. A006-2-1) levels were measured using commercial kits from Nanjing Jiancheng Bioengineering Institute. Iron levels were determined using an iron assay kit (Cat. ab83366, Abcam). All procedures were performed strictly following the manufacturers’ instructions.

### Western blot

For protein extraction, LX-2 cells were lysed using RIPA lysis buffer (Beyotime, Shanghai, China). Total protein concentration was determined using a BCA Protein Assay Kit (Beyotime). Protein aliquots were separated by 10% SDS-PAGE, electrotransferred onto PVDF membranes (Millipore, USA), and blocked with 5% skim milk for 1 h. The membranes were then incubated with primary antibodies at 4 °C for 12 h, followed by incubation with secondary antibodies for 2 h at room temperature. Protein bands were visualized using an ECL detection kit (Millipore). Primary antibodies were obtained from Abcam, including anti-KAT2A (dilution 1:1500), anti-KAT3B (1:1200), anti-CPT1A (1:1500), anti-SIRT5 (1:1000), anti-SIRT7 (1:1200), anti-Collagen I (1:1000), anti-Fibronectin (1:1800), anti-α-SMA (1:1000), anti-GPX4 (1:1600), anti-SLC7A11 (1:1200), anti-p53 (1:600), anti-ACSL4 (1:1000), and anti-GAPDH (1:3000). The anti-succinylation antibody (1:1000) was purchased from PTMBIO. GAPDH was used as a loading control to normalize protein expression.

### Co-immunoprecipitation (Co-IP) assay

Briefly, LX-2 cells were lysed on ice and centrifuged at 17,000×*g* for 15 min. The supernatant was collected, and immunoprecipitation was performed using Protein A/G-agarose beads (Thermo Fisher Scientific) with anti-SIRT5 or anti-GPX4 antibodies, using IgG as a negative control. The precipitated fractions were eluted from the beads and analyzed by Western blot.

### GPX4 succinylation site prediction and mutation

The GPSuc website (http://kurata14.bio.kyutech.ac.jp/GPSuc/index.php) was used to predict succinylation sites of GPX4. Arginine (R) mutations were introduced at lysine (K) residues K117, K148, K152, and K162 of GPX4 (yielding K117R, K148R, K152R, and K162R mutants). Wild-type (WT) GPX4 and the mutants were separately transfected into LX-2 cells for 24 h. All constructs were designed and synthesized by Genscript Biotechnology Co., Ltd.

### Protein stability assessment

To evaluate GPX4 protein stability following SIRT5 knockdown, LX-2 cells were treated with cycloheximide (CHX; 100 μg/ml; Abcam), a protein translation inhibitor. GPX4 protein levels were then detected at different time points (0, 6, 12, 18, and 24 h).

### Animal experiment

The animal experimental protocol was approved by the Ethics Committee of Guiyang Healthcare Vocational University (Approval No. 2023101, dated August 15, 2023). This study was conducted in full compliance with the ARRIVE guidelines 2.0 (Animal Research: Reporting of In Vivo Experiments) for reporting animal research. All animal experiments were performed in accordance with the National Institutes of Health Guide for the Care and Use of Laboratory Animals (eighth edition, 2011) and the relevant guidelines and regulations of China.

A total of 36 eight-week-old male C57BL/6 mice (body weight 20 ± 2 g) were provided by Beijing Charles River Experimental Animal Technology Co., Ltd (Certificate No. SCXK 2022-0001). All animals were housed under specific pathogen-free conditions with a 12 h light/dark cycle (lights on 7:00 AM–7:00 PM), controlled temperature (22 ± 2 °C), and relative humidity (50 ± 10%). Food and water were available ad libitum. After 1 week of acclimatization, the mice were randomly assigned to six groups (n = 6 per group) using a computer-generated random number sequence (Random Allocation Software v2.0): Control group, CCl_4_ alone group, CCl_4_ + sh-NC group, CCl_4_ + sh-SIRT5 group, CCl_4_ + sh-SIRT5 + lv-NC group, and CCl_4_ + sh-SIRT5 + lv-GPX4 group. Hepatic fibrosis was induced by intraperitoneal (i.p.) injection of 10% CCl_4_ in olive oil at a dose of 10 ml/kg body weight twice per week (every 3–4 days, specifically on Monday and Thursday) for eight consecutive weeks (34). The Control group received an equal volume of olive oil alone administered at the same frequency.The sample size (n = 6 per group) was determined *a priori* based on a power analysis using pilot data for serum ALT level as the primary outcome measure (expected effect size = 1.5, α = 0.05, β = 0.20; calculated using G∗Power 3.1). No animals were lost or excluded from the analysis; no predefined exclusion criteria were applied.

For SIRT5 knockdown, a lentiviral vector (pLVX-shRNA1, Clontech, USA) expressing a short hairpin RNA targeting SIRT5 (sh-SIRT5) was constructed. A non-specific shRNA (sh-NC) served as the negative control. sh-SIRT5: 5′-GCACTGTTGCCGAGAACTATA-3′; sh-NC: 5′-CCTAAGGTTAAGTCGCCCTCG-3′. For GPX4 overexpression, the full-length human GPX4 coding sequence was subcloned into a pLVX-Puro lentiviral vector (Clontech). The lentiviral particles were packaged in HEK293T cells using standard protocols and concentrated by ultracentrifugation to a final titer of 1 × 10^9^ TU/ml. All lentiviral injections were administered *via* tail vein injection in a volume of 200 μl PBS containing:

For sh-SIRT5 or sh-NC: 2 mg/kg (based on shRNA plasmid weight); or.

For lv-GPX4 or lv-NC: 2 × 10^7^ transduction units per mouse.

The first injection was given 4 h prior to the first CCl_4_ treatment, and subsequent injections were repeated once weekly (on the day before the first CCl_4_ injection of the week) for the entire 8-weeks experimental period. This schedule was chosen to maintain sustained SIRT5 knockdown and GPX4 overexpression throughout the fibrosis induction.

For all surgical and terminal procedures, mice were anesthetized by inhalation of isoflurane (induction: 4%, maintenance: 1.5–2% in oxygen) using a precision vaporizer (RWD Life Science). Euthanasia was performed by cervical dislocation under deep isoflurane anesthesia, in accordance with the AVMA Guidelines for the Euthanasia of Animals (2020 edition). All outcome assessments—including histological staining (HE, Masson, Sirius Red) and serum biochemical measurements (AST, ALT, ALP)—were performed by investigators blinded to the group allocation. After the 8-weeks treatment period, all mice were euthanized as described; serum and liver tissues were collected. Serum AST, ALT and ALP levels were measured using commercial kits (Nanjing Jiangcheng Bioengineering Institute). Livers were weighed, used to detect the hydroxyproline levels using the commercial kits provided by Nanjing Jiangcheng Bioengineering Institute fixed in 4% paraformaldehyde, and paraffin-embedded for H&E, Masson and Sirius Red staining to evaluate liver histomorphology. The animal experimental protocol was approved by the Ethics Committee of Guiyang Healthcare Vocational University (Approval No. 2023101).

### Statistical analysis

All results were expressed as mean ± standard deviation. The statistical analysis was performed using SPSS 20.0 software (IBM Corp.). All experiments were conducted independently three times. Meanwhile, one-way ANOVA followed by Tukey was applied to assess differences between multiple groups. A statistically significant difference was set at *p* < 0.05.

## Ethics approval and consent to participate

The animal experimental protocol was approved by the Ethics Committee of Guiyang Healthcare Vocational University (Approval No. 2023101, dated August 15, 2023). This study was conducted in full compliance with the ARRIVE guidelines 2.0 (Animal Research: Reporting of In Vivo Experiments) for reporting animal research. All animal experiments were performed in accordance with the National Institutes of Health Guide for the Care and Use of Laboratory Animals (eighth edition, 2011) and the relevant guidelines and regulations of China.

## Data availability

The datasets used and/or analyzed during the current study are available from the corresponding author on reasonable request.

## Supporting information

This article contains supporting information.

## Conflict of interest

The authors declare that they have no conflicts of interest with the contents of this article.
