# Supplementary material for: Sirtuin five knockdown induced the ferroptosis of hepatic stellate cells in hepatic fibrosis progression through de-succinylation modification of GPX4
Source: J Biol Chem. 2026 Jul 24;302(9):113366. doi: 10.1016/j.jbc.2026.113366 (PMC13524596; doi:10.1016/j.jbc.2026.113366)
Supplement: Supplementary file 1 [file mmc1.docx]

**Supplementary Figure 1 Ponceau S staining**

The Ponceau S staining was used as a loading control for the global succinylation.

**Supplementary Figure 2 SIRT5 knockdown inhibited the activation of LX-2 cell by inducing the ferroptosis**

The LX-2 cells were treated with 5 ng/mL TGF-β1 and tansfected with sh-SIRT5 2#. (A) The cell viability was detected by CCK-8 assay. The Iron (B), MDA (C), ROS (D) and GSH (E) levels were detected by commercial kits. (F-I) The protein levels of Collagen I, fibronectin, and α‐SMA were detected by western blot. **P<0.01 vs Control group. #P<0.05, ##P<0.01 vs TGF-β1+sh-NC group. Each experiment has three biological replicates and three technical replicates.

**Supplementary Figure 3 K162 mutation of GPX4 inhibited the activation of LX-2 cell by inducing the ferroptosis**

The LX-2 cells were transfected with SIRT5 overexpression vector, wild type and K162 mutation type of GPX4. The mRNA and protein levels of SIRT5 in the LX-2 cells were detected by RT-qPCR (A) and western blot (B and C) assays. (D) The cell viability was detected by CCK-8 assay. The Iron (E), MDA (F), ROS (G) and GSH (H) levels were detected by commercial kits. (I-L) The protein levels of Collagen I, fibronectin, and α‐SMA were detected by western blot. **P<0.01 vs oe-NC group. #P<0.05, ##P<0.01 vs oe-SIRT5 group. &P<0.05, &&P<0.01 vs oe-SIRT5+WT group. Each experiment has three biological replicates and three technical replicates.
